# Identification of Candidate Genes and Pathways Linked to the Temperament Trait in Sheep

**DOI:** 10.3390/genes15020229

**Published:** 2024-02-11

**Authors:** Estefanía Romaniuk, Brenda Vera, Pablo Peraza, Gabriel Ciappesoni, Juan Pablo Damián, Elize Van Lier

**Affiliations:** 1 Departamento de Producción Animal y Pasturas, Facultad de Agronomía, Universidad de la República, Avda. Garzón 780, Montevideo 12900, Uruguay; romaniuk@fagro.edu.uy; 2 Estación Experimental Facultad de Agronomía Salto, Ruta 31, km 21, Salto 50000, Uruguay; 3 Sistema Ganadero Extensivo, Instituto Nacional de Investigación Agropecuaria, INIA Las Brujas, Ruta 48, km 10, Canelones 90200, Uruguay; bvera@inia.org.uy (B.V.); pperaza@inia.org.uy (P.P.); gciappesoni@inia.org.uy (G.C.); 4 Departamento de Biociencias Veterinarias, Facultad de Veterinaria, Universidad de la República, Ruta 8, km 18, Montevideo 13000, Uruguay; jpablodamian@gmail.com; 5 Núcleo de Bienestar Animal, Facultad de Veterinaria, Universidad de la República, Ruta 8, km 18, Montevideo 13000, Uruguay

**Keywords:** ssGWAS, ovis aries, *PYMG*, *CAPN1*, *GRID2*, *SYT7*

## Abstract

Temperament can be defined as the emotional variability among animals of the same species in response to the same stimulus, grouping animals by their reactivity as nervous, intermediate, or calm. Our goal was to identify genomic regions with the temperament phenotype measured by the Isolation Box Test (IBT) by single-step genome-wide association studies (ssGWAS). The database consisted of 4317 animals with temperament records, and 1697 genotyped animals with 38,268 effective Single Nucleotide Polymorphism (SNP) after quality control. We identified three genomic regions that explained the greatest percentage of the genetic variance, resulting in 25 SNP associated with candidate genes on chromosomes 6, 10, and 21. A total of nine candidate genes are reported for the temperament trait, which is: *PYGM*, *SYVN1*, *CAPN1*, *FADS1*, *SYT7*, *GRID2*, *GPRIN3*, *EEF1A1* and *FRY*, linked to the energetic activity of the organism, synaptic transmission, meat tenderness, and calcium associated activities. This is the first study to identify these genetic variants associated with temperament in sheep, which could be used as molecular markers in future behavioral research.

## 1. Introduction

Temperament can be used for understanding animal behavior and can be defined as the emotional variability among animals of the same species in response to the same stimulus [1]. Animals perceive environmental stimuli, such as novelty, uncertainty, challenge, or change, and may react in different ways [2]. Temperament refers to the individuals’ consistent behavioral style or tendency and can be represented in opposites like shy or bold, sociable or aggressive, restless or quiet [3]. Different physiological and behavioral responses have been used as indicators to classify animals according to their temperament [4], and can be described as calm, nervous, or neutral. Temperament can influence productive traits such as ovulatory rate [5,6], meat quality [7], animal weight gain [8], milk and colostrum quality [9,10], favoring animal welfare [11] and providing safety to the operator when associated with less reactive animals or of a calmer temperament [12].

Variability in animal temperament may be influenced by polymorphism of genes that are expressed in the brain, or along the sympathetic-adrenal-medullary axis (SAM) of the autonomic nervous system and hypothalamus-pituitary-adrenal axis (HPA) [13,14]. The Single Nucleotide Polymorphism (SNP) is the most common type of polymorphism where a single base is changed for another (e.g., GGTACC/GGTGCC) at a frequency greater than 1% in a population [15]. Since 1990, the University of Western Australia (UWA) has selected Merino sheep establishing lines of calm and nervous sheep [14]. In this temperament flock, eight SNP were found distributed differently between calm and nervous sheep [16] and associated with the serotonin, oxytocin, and dopamine systems as well as the stress response axis [13,16]. A recent review [17] of genomic regions and candidate genes about behavioral traits, reported 148 genomic regions associated with 22 behavioral traits [13,18,19] and 15 candidate genes in sheep.

The working hypothesis of this study was that there are SNP associated with the temperament trait in Australian Merino sheep. The main objective was to identify the SNP associated with the temperament phenotype that explained the greatest genetic variance and to subsequently do gene-set analyses to identify candidate genes, functional gene sets, and gene signaling pathways implicated in the temperament of sheep.

## 2. Materials and Methods

### 2.1. Phenotypic and Pedigree Data

A total of 4317 lambs were tested for temperament between one and three months post-weaning, including four progenies: 2010, 2011, 2018, and 2019. The records of temperament scores were included in the genetic database (SULAR, Uniform System of Survey and Records Storage, of the National Genetic Evaluation). Temperament was measured using the Isolation Box Test (IBT) [20,21]. The dimensions of the box were 1.50 m (L) × 1.50 m (H) × 0.75 m (W). Each lamb was gently pushed inside the box and held there for 30 s, and agitation was objectively measured by an agitation meter. The agitation meter registered the vibrations of the box induced by the lamb’s movements and high-pitched vocalizations. The higher the agitation score, the more nervous the sheep. The average age of the lambs at IBT was 160 ± 38 days. The IBT was calibrated with an electronic unit to high and low agitation scores. The intra-test coefficients of variation (CV %) of the IBT were 7.17% and 7.93% for the high and low settings, respectively. The inter-test CV % of the IBT were 9.37% and 9.29% for the high and low settings, respectively. There was no previous selection of animals and they had no previous experience with the IBT. 

Genealogical information from 10,799 animals was provided by the Rural Association of Uruguay and the Merino Breeders Society. The genotyped animals were checked for parent–offspring incompatibilities based on Mendelian conflict counts using the SeekParentF90 software, version 1.52 [22]. A Pearson correlation was performed between the off-diagonal elements of the genomic and pedigree information matrices to verify the consistency of the data.

### 2.2. Genotypic Data and Quality Control

DNA was extracted from blood samples following the protocol described by Medrano et al. [23]. A total of 1697 animals were genotyped for 43,705 SNP and the molecular information was obtained using the Geneseek Genotyping Profile panel (GeneSeek ^®^ Genotyping Profile, GGP, Illumina, San Diego, CA, USA). Genomic data quality control (QC) was conducted using PREGSF90 version 1.23 [22] excluding markers located in sexual chromosomes, monomorphic SNP, with minor allele frequencies (MAF) < 0.05 and call rates <85%. Animals were removed from the analysis when call rates were <90%. A total of 38,268 effective SNP were retained for subsequent genomic analyses.

### 2.3. Model and Estimation of Genetic Parameters

The mixed model to perform a ssGWAS included fixed effects and random effects. The fixed effects were contemporary group (year, flock, sex, and management group), dam age (<2 years, 2 to 3 years, or >3 years), and type of birth (single or twin). The age of the lamb at the time of measurement with the IBT was considered a covariate. The SNP were incorporated into the model as random effects.

The following model was used (Zambra et al. [24]):

y_ijkml_ = CG_i_ + tb_j_ + ad_k_ + βla_ijkm_ + am + e_ijklm_

where y is the temperament data of the lamb m; CG_i_ is the contemporary group (i: year-flock-sex-management group; 73 levels), tb_j_ the type of birth of lamb (j: single (1), twin (2)), ad_k_ the age of dam subdivided into 3 classes (k_1_ = < 2 years ewe, k_2_ = 2 to 3 years ewe and k_3_ = > 3 years ewe), β is the regression coefficient of the age of the lamb on temperament, la is the covariate of lamb age (in days) at the time of measurement, a is the additive effect of the animal with distribution am ~ N (0, σ^2^_A_ A) where σ^2^_A_ is the additive variance of temperament, A is an additive relationship matrix, and e is the random effect of the error with independent normal distribution between the observations with variance σ^2^e.

The variance components necessary for the model were estimated by airemlf90 program version 1.148 of BLUPF90.

The heritability was calculated as:

$$h^{2}=\frac{\sigma_{a}^{2}}{\left(\sigma_{a}^{2}+\sigma_{e}^{2}\right)}$$

where $\sigma_{a}^{2}$ is the genetic variance and $\sigma_{e}^{2}$ is the residual variance.

### 2.4. Single Step Genome-Wide Association Studies (ssGWAS)

The ssGWAS methodology allows genome-wide association studies combining all available pedigree, phenotype, and genotype information in a single evaluation using the BLUPf90 family of programs [25]. The result of combining the genomic (SNP) and pedigree information is the [H] matrix, where the molecular information of genotyped animals is projected, through relatives, onto individuals that are not genotyped [22]. Here, the classical pedigree relationship [A] matrix is replaced by the [H] matrix. Thus, all the SNP are considered simultaneously together with all the phenotypes of the genotyped and non-genotyped animals [22,26]. The [H] matrix is complex, it can be simplified to its inverse [H^−1^] [27]:

$$H^{-1}=A^{-1}+\left[\begin{array}{cc} 0 & 0\\ 0 & G^{-1}-A_{22}^{-1} \end{array}\right]$$

where G^−1^ is the inverse of the genomic relationship matrix (genotyped animals, proportion of alleles shared between animals); A^−1^ is the inverse of the pedigree relationship matrix; and A_22_^−1^ is the inverse of the pedigree relationship matrix for genotyped animals. The [G] matrix was computed as [28]:

$$G=\frac{ZDZ’}{\sum_{i=1}^{M}2p_{i}(1-p_{i})}$$

where Z is an incidence matrix adjusted for allele frequencies; D is a diagonal matrix of weights for SNP variances; Z’ is the transpose; M is the number of SNP; and p_i_ represents the minor allele frequency of each SNP.

The percentage of genetic variance (% var) explained by a region has been calculated as follows [22]:

$$\frac{Var\left(a_{i}\right)}{\sigma_{a}^{2}}\times 100\%=\frac{Var(\sum_{j=1}^{B}Z_{jûj})}{\sigma_{a}^{2}}\times 100\%$$

where a_i_ is the genetic value of the i-th region; B is the total number of adjacent SNP within a 0.5 Mb region; $\sigma_{a}^{2}$ is the total genetic variance; Z_j_ is the vector of the gene content of the j-th SNP for all individuals, and û_j_ is the marker effect of the j-th SNP within the i-th region.

The SNP effects were estimated according to Stranden and Garrik [29]:

$$â=\frac{1}{2\sum p_{i}q_{i}}Z’G^{-1}{û}_{2}$$

where Z’ is the transpose of Z matrix; G is the genomic matrix for the genotyped animal; ${û}_{2}$ is the genomic breeding value for genotyped animals and p_i_q_i_ are the allele frequencies.

### 2.5. Identification of Positional Candidate Genes

The threshold of non-overlapping windows that explained more than 0.5% genetic variance [30,31] were reported and were considered candidate regions to verify overlapping genes. The SNP that explained a greater genetic variance in the window was reported. Manhattan plots based on the proportion of additive genetic variance explained by the windows were generated using the R.Script output from POSTGSF90 version 1.75 [25].

## 3. Gene Enrichment Analysis

On the other hand, and only for this approach, the 5% of SNP with the greatest effect were selected [29,31]. An SNP was assigned to a gene if it was located inside a gene or within a genomic distance of 5 kb upstream or downstream from a gene [30,31]. To assign SNP to genes, computational procedures were used with an ad hoc script in the R programmer (version 4.1.0) gene locations were obtained using the BiomaRt package (version 2.48.3) [32,33] and the Ovis_aries_v3.1 ovine reference genome data set (https://www.ensembl.org; accessed on 4 April 2022 [34]). Therefore, a gene was associated with temperament if it contained at least one SNP whose effect was in the top 5% of the distribution. The Database for Annotation, Visualization and Integrated Discovery (DAVID, latest available version 6.8) was used to assign the genes to functional categories [35]. The objective of this analysis was to know in which metabolic pathways the genes would be involved and to quantify them through the enriched *p*-value. The available Gene Ontology (GO) terms for molecular function (MF), cellular component (CC), and biological process (BP) were included in the analysis, and the Kyoto Encyclopedia of Genes and Genomes (KEGG) databases were used to reveal the functional implications of the detected genes. Finally, the significance of the enriched terms was a threshold *p*-value ≤ 0.05, as implemented in the DAVID platform, available version 6.8.

## 4. Results

### 4.1. Estimation of Genetic Parameters

The genetic variance was 111.8 ± 22.8 (SEM) and the residual variance was 472.6 ± 20.80 (SEM). The heritability (h^2^) of the temperament trait was 0.19 ± 0.038 (SEM).

### 4.2. Single Step Genome-Wide Association Studies (ssGWAS)

**Figure 1:**
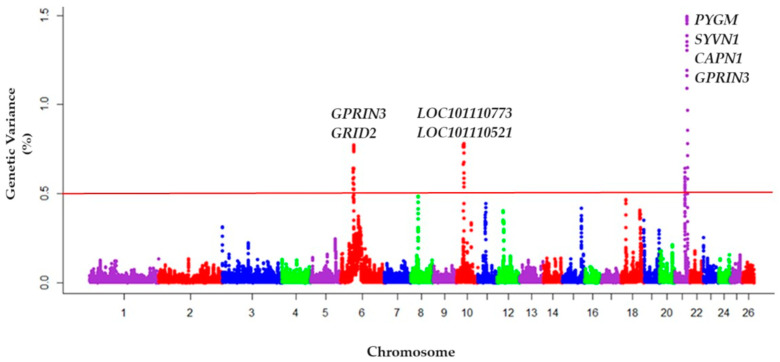
Manhattan plot of 38,268 effective SNP for 1697 Australian Merino genotyped animals belonging to the National Genetic Evaluation, included in the database of the SULAR. Chromosome number and statistical significance threshold according to the % genetic variance explained by windows variance of 0.5 mbps. The solid horizontal line is the threshold, % var ≥ 0.5%. The nine candidate genes involved in the temperament trait on chromosomes 6, 10, and 21 were inserted with their respective codes within each chromosome.

The genetic variance explained by windows of 0.5 mbp of adjacent SNP, identified genomic regions of interest associated with chromosomes 6, 10, and 21 (var ≥ 0.5) (Figure 1).

The information on the most relevant SNP found in these regions showed that there were nine candidate genes for the temperament trait (Table 1), which were: Glycogen Phosphorylase, Muscle associated gene (*PYGM*) (21:42,295,599–42,307,126), Synoviolin 1 gene (*SYVN1*) (21:42,650,559–42,655,527), Calpain subunit 1 gene (*CAPN1*) (21:42,712,976–42,740,799), *LOC101110773* gene (10:29,275,771–29,457,586), *LOC101110521* gene (10:28,986,741–29,188,660), Glutamate Ionotropic Receptor Delta subunit 2 gene (*GRID2*) (6:30,768,380–31,534,647), Fatty Acid Desaturase 1 gene (*FADS1*) (21:39,652,537–39,665,108), Synaptotagmin 7 gene (*SYT7*) (21:39,390,965–39,426,334) and the G Protein-Regulated Inducer of Neurite outgrowth 3 gene (*GPRIN3*) (6:35,511,293–35,513,635). The genomic regions of chromosomes with adjacent SNP explaining more than 0.5% of the additive genetic variance, are reported in Table 2. On chromosome 21, the variant rs402505013 (21:42,295,749) determined the greatest genetic variance (1.46%) and five candidate genes were detected. On chromosomes 6 and 10, two candidate genes each were detected.

### 4.3. Enrichment Analysis

**Figure 2:**
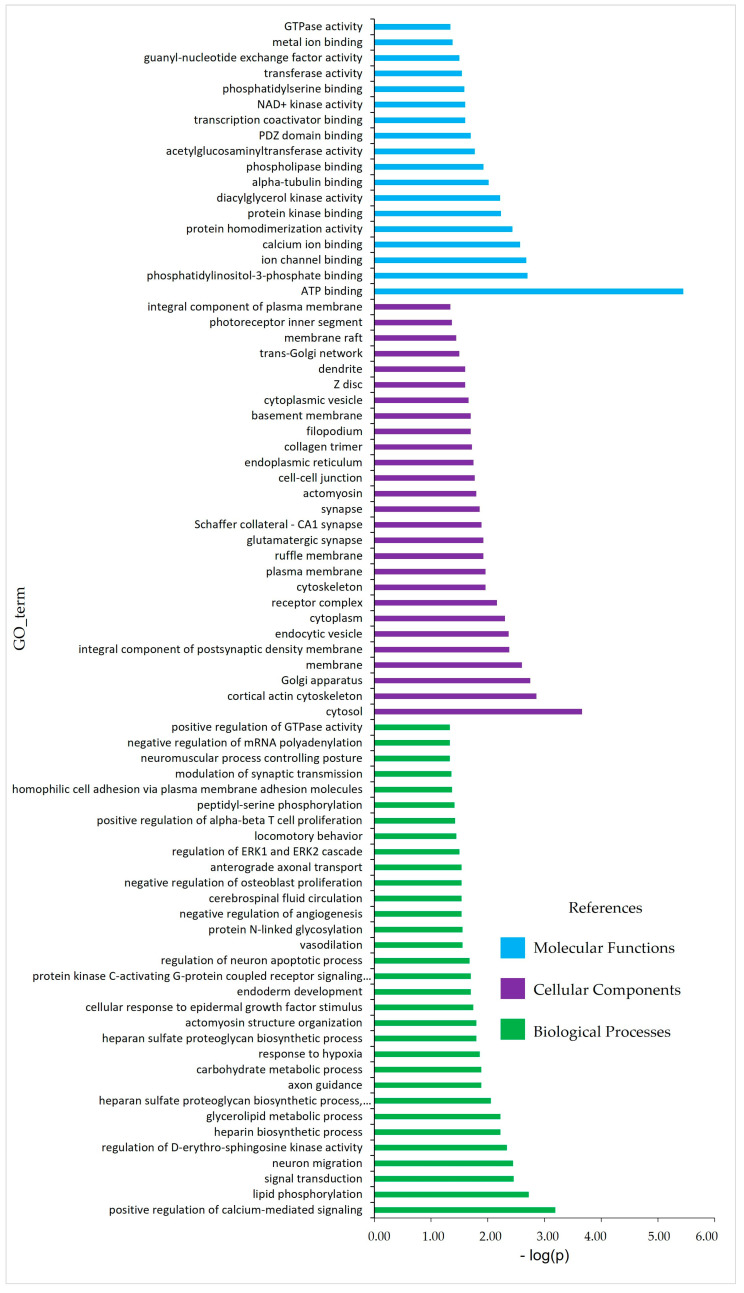
Gene ontology categories represented by Molecular Functions (light blue bars), Cellular Components (purple bars), and Biological Processes (green bars), for the set of positional candidate genes with the 5% of SNP with the greatest effect in function del - log of the enriched *p*-value.

For the enrichment analysis, 2185 SNP were considered (5% SNP greatest effect). These SNP defined a set of 900 genes in the sheep reference genome. The enriched *p*-value of each biological term indicated the importance of the term with respect to the set of analyzed genes (Table 3, Figure 2). Several significant metabolic pathways (*p*-value ≤ 0.05) were identified and grouped into four categories: signaling, metabolism, steroidogenesis, and others (Table 3). The gene ontology of functional enrichment is shown in Figure 2. There were several terms linked to the regulation of ATP, calcium, cell activity, locomotion and behavior, lipid and carbohydrate metabolism, modulation of synaptic transmission, and GTPase activity, among others.

## 5. Discussion

The hypothesis that there are SNP associated with the temperament trait in Australian Merino sheep was confirmed. The genetic variance of the SNP showed that there are several regions associated with greater variability, suggesting that temperament is not solely governed by the effect of one major gene. Instead, it appears to be a complex multigenic trait, influenced by multiple genes distributed across the genome, as suggested by Hazard et al. [36]. As part of the results of this study, nine positional candidate genes and gene signaling pathways underlying temperament in the Australian Merino sheep were detected, several of which have not been previously reported. These findings are valuable and relevant due to the following: (1) we worked with a population of animals that were not previously selected for temperament, (2) we utilized a molecular panel and information that included data reported by groups of Australian, European, and American researchers, and (3) the size of the sample of genotyped animals and the genealogy was appropriate for the methodology used. Therefore, the source of information to do the association study was reliable and robust and considered the structure of the population by including the genotype and genealogy matrices.

### 5.1. Heritability (h^2^)

The estimated heritability of temperament was 0.19 ± 0.038, which is a moderate h^2^ [37]. The h^2^ value shows that it would be feasible to include the temperament trait in a selection program. The estimated value (0.19) is consistent with the h^2^ for adult ewes (0.20) [38] and is similar to the h^2^ for Corriedale lambs (0.18) [24]. In another sheep study considering a subpopulation of the phenotypic data used in the present study, the h^2^ was 0.31 ± 0.06 (two generations, n = 2952 animals) [24]. The difference in the estimated h^2^ value may be due to an increase in the number of measured individuals and some of the new animals are offspring of those that had been measured before. In the study by Zambra et al. [24] there were no measurements of parents/offspring with data. Additionally, the inclusion of genomic information from the animals translates into higher precision. On the other hand, in an Australian report that included more sheep breeds, the h^2^ was higher: Australian Merino 0.38; Poll Dorset 0.41; White Suffolk 0.29; and Poll Merino 0.41 [20]. The h^2^ for other productive species is similar to our estimated value (cattle: 0.22–0.26, [39,40]; horses: 0.23, [41]). Even though temperament is not economically valued, several studies confirm its incidence in productive traits [1,6,7,8,9,10].

### 5.2. Chromosomes and Candidate Genes

In chromosome 21, one SNP defined the greatest genetic variance and is within the *PYMG* gene (21:42,295,599–42,307,126) encoding the enzyme muscle glycogen phosphorylase, an allosteric enzyme that plays a central role in maintaining cellular and body glucose homeostasis [42]. A study about protein phosphorylation levels with different meat tenderness post-mortem, shows that it is the main protein involved in the regulation of energy metabolism and reported a positive correlation between the phosphorylation level of glycogen phosphorylase and the rate of glycolysis [43,44,45]. In stressful situations such as social isolation, stress hormones like adrenaline, stimulate glycogen phosphorylase to quickly release glucose from muscle glycogen. The deficiency of this enzyme has been reported in humans and sheep as the cause of symptoms of intolerance to exercise since less glucose would be available for muscle contraction [42,46,47]. Glycogen phosphorylase is one of the most important enzymes involved in the tenderness of meat and may affect the quality of it [48]. Therefore, genetic variations could not only affect what is related to stress or welfare, but the stress conditions linked to the end of the productive stage will determine the characteristics of the final product, in this case, the meat [48,49].

Variants rs413708295 (21:42,714,381), rs421553713 (21:42,714,613) and rs161627624 (21:42,715,850) identified *CAPN1* gene (21:42,712,976–42,740,799). This gene encodes the μ-calpain 1, a proteolytic enzyme with activity on myofibrillar proteins [50,51,52]. This is associated with proteolytic proteins directly linked to the tenderness of the meat and that is essential for postmortem proteolysis in the process of transforming muscle into meat [50]. Variants in the *CAPN1* gene were associated with weaning weight (rs417258958), rib eye area (rs403953588 and rs430307080), fat thickness (rs408790217), body depth (rs420860201), and heights at croup and withers (rs408790217) [53], all of these traits were recorded in vivo. Those variants in these genes were also associated with physicochemical meat traits such as pH, color, tenderness, and water-holding capacity [54]. The differential gene expression of *CAPN1* among animals of different temperaments might be involved in a lower meat quality for the more reactive animals, which needs further research for confirmation.

The rs161627521 (21:42,654,067) variant is within the *SYVN1* gene (21:42,650,559–42,655,527). The *SYVN1* gene has been extensively studied in relation to body weight regulation and mitochondrial biogenesis. To our knowledge, this is the first study documenting variants in the *SYVN1* gene in sheep. More studies are necessary to understand the implications of this gene in sheep and their productive traits. Another gene identified by two markers rs403363266 (21:39,653,383) and rs427110197 (21:39,654,860), was the *FADS1* gene (21:39,652,537–39,665,108), associated with the W5PWA9 protein in sheep [55] and as a member of the fatty acid dehydrogenase family it is related to all lipids [56]. The last candidate gene associated with the variant rs421709693 (21:39,432,569) was *SYT7* gene (21:39,390,965–39,426,334). The *SYT7* gene has been studied in relation to synaptic transmission [57] and behavioral abnormalities [58]. In terms of temperament, there is limited research specifically linking the *SYT7* gene to this trait. Mice with silenced genes, including *SYT7* in the hippocampus, showed manic-like and depressive-like behavioral fluctuations, which were analogous to the mood cycling symptoms of bipolar disorder, suggesting that *SYT7* may be a candidate risk factor for behavioral abnormalities [59]. The molecular functions and biological processes linked to this gene are associated with calcium (e.g., regulation, dependent activation for the fusion of synaptic vesicles, exocytosis of neurotransmitters, repair of the plasmatic membrane, regulation of dopamine secretion, glucagon, and insulin secretion, among others) [55]. The main enzymes of proteolysis depend on calcium [60] and anything related to plasma calcium concentrations could be involved in meat quality. On the other hand, there are genes that have been associated with behavioral traits and that are also found within or near the genomic region of interest on chromosome 21, such as the Muscarinic Cholinergic Receptor (*CHRM1*) gene (21:37.9–48.4 Mb), which would be linked to locomotion, cognition, and the nervous system [61]. Therefore, the SNP of chromosome 21 found in the present study could be used as molecular markers for future studies focused on candidate genes associated with temperament or behavioral traits that affect reactivity and that are associated with meat quality.

The variants rs408317317 (10:29,35”,089’, rs422288687 (10:29,304,176), rs400430030 (10:29,421,760), rs419203432 (10:29,415,140), and rs398157763 (10:29,455,959) identified the gene LOC101110773 (*EEF1A1*) (10:29,275,771–29,457,586) on chromosome 10, linked to molecular functions of energy activity. Specifically, the encoded protein W5PD15 (elongation factor α 1) promotes GTP-dependent binding of aminoacyl-tRNA to the A-site of ribosomes during protein biosynthesis [55]. Another gene detected was the LOC101110521 (10:28,986,741–29,188,660). The five associated markers were rs427220269 (10:29054709), rs421383362 (10:29,188,403), rs4098299992 (10:29,162,222), and rs419116702 (10:29,030,595). This gene on the Ensembl platform is described as the *FRY* gene. A phenotypic study in Merino and Merino-derived sheep reported this gene as one of the major signals in the genome linked to the phenotypic traits of wool [62]. In this same study, the *EEF1A1* gene (LOC101110773) was also described as part of a set of genes of biological interest that contributes to elucidating the genetic basis of the Merino phenotype. Two SNP have been reported for the temperament trait [16] in Merino sheep and the associated gene is 5–hydroxytryptamina receptor 2A (HTR2A) in chromosome 10 (rs17196799, rs7193181) [16]. Half of the SNP of chromosome 10 that contribute to the genetic variance of temperament were associated with molecular functions of energy activity. This is consistent with aspects of reactivity, such as the physiological response to stress, where the animal recognizes the threat to homeostasis and biological responses are activated to restore normal homeostasis function and welfare [63].

On chromosome 6 the *GPRIN3* candidate gene (6:35,511,293–35,513,635) associated with the variants rs111759303 (6:35,511,497), rs424142667 (6:35,511,899) and rs403382565 (6:35,511,899) was identified. This gene is highly expressed in the adrenal gland and codes for the W5NQE9 protein, but actually, there is not a lot of information about it [64]. How the *GPRIN3* gene is expressed in the adrenal gland, is a relevant finding given that the adrenal gland is involved in the stress response (SAM and HPA axes, [63,65]) and there are differences in the frequency of polymorphism according to the type of temperament [13]. The A/A genotype of the SNP628 type polymorphism of the *CYP17* gene specifically involved in cortisol production was more frequent in sheep selected for nervous temperament, while the G/G genotype was in sheep selected for calm temperament [13].

The other candidate gene on chromosome 6 is *GRID2* (6:30,768,380–31,534,647) associated with two markers, rs399480023 (6:31,217,615) and rs422603241 (6:31,453,177). This gene codes for the W5QA32 protein (glutamate receptor), which is related to biological processes linked to the regulation of synaptic transmission [55]. Glutamate is an essential amino acid and is the most abundant neurotransmitter in the brain, also involved in the regulation of behavioral, social, learning, memory, sensory, and cognitive processes [66]. Glutamate regulates sexual behavior through dopamine, due to its action on hypothalamic GnRH neurons [67]. Recent research in sheep demonstrated that the inclusion of specific amino acids in the diet, such as arginine, glutamine, leucine, and glycine, has beneficial effects on embryonic and fetal survival and growth [68,69]. On the other hand, a study to identify QTL (Quantitative Trait Locus) for behavioral reactivity revealed a QTL that maps within the gene (Glutamate receptor metabotropic 7), associated with locomotion in response to social isolation [36]. In addition, this QTL is close to the gene encoding the oxytocin receptor (OXTR) which is associated with social [70] and maternal behaviors [71,72]. A study supports this, revealing an SNP rs17664565 in chromosome 19 maps within the gene OXTR [16]. Therefore, the SNP detected on chromosome 6 identified two relevant genes, one that is expressed in the axis linked to the stress response (HPA axis, *GPRIN3* gene) and another that is expressed centrally (*GRID2* gene) associated with behavioral traits. Temperament is key in both scenarios since greater reactivity negatively affects the functioning of the reproductive endocrine axis [73,74].

### 5.3. Functional Enrichment Analysis

Enrichment analysis revealed several functional pathways and biological processes associated with the temperament trait. The gene-set is associated with a set of annotation terms. If the genes share a similar set of those terms, they are most likely involved in similar biological mechanisms. The DAVID platform provides an algorithm that adopts Kappa statistics, which enables the quantitative measurement of the degree of agreement of genes that share similar annotation terms. The Kappa result ranges from zero to one (higher value, higher agreement). In the calcium signaling pathway (*p*-val = 0.048) several genes were grouped, but the most important were: *HTR5A* gene, serotonin receptor; *DRD1* gene, dopamine receptor, and *GRM1* gene, glutamate receptor 1. A more detailed exploration of calcium signaling was associated with the following annotation terms: cardiac muscle contraction (kappa = 0.42); oxytocin signaling (kappa = 0.41); glutamate synapse (kappa = 0.35) and dopamine synapse (kappa = 0.31). The neurotransmitters dopamine and serotonin are involved in the stress response in a variety of species, through the activation of their different receptors [75,76]. Oxytocin is an important regulator of social behaviors [70] such as maternal behavior [77] and recognition in humans [78], and polymorphism in the oxytocin receptor gene has been associated with temperament, reactivity to stressors, and aggressive behaviors [79,80]. Therefore, the gene-set revealed that the enriched calcium signaling pathway is closely related to temperament because involves many aspects of this trait and its physiological effects, as has been shown previously in reproductive aspects [5,6].

Five genes were identified in the metabolic pathway of steroidogenesis in the ovary (*p*-val = 0.0170): *CYP2J* gene (cytochrome P450); follicle-stimulating hormone receptor gene (*FSHR*); gene for the enzyme hydroxysteroid 17-β dehydrogenase (*HSD17B2*) and two genes associated with prostaglandin synthesis (*PGFS*, *PTGS2*). These genes linked to reproduction reaffirm the association of temperament with reproduction and its effect on the reproductive endocrine axis (hypothalamic-pituitary-gonadal, HPG, axis) [74,81].

In the metabolic pathways, the *ACTN2* gene was found to be associated with arrhythmogenic cardiomyopathy of the right ventricular (*p*-val = 0.037). This gene codes for the protein actinin α 2, reported for humans, which is expressed in both skeletal muscles and the heart, and its function is the anchoring of thin myofibrillar actin filaments [82,83]. A variant in the regulatory region of this gene leads to heart failure [84]. However, to our knowledge, this is the first report of its association with the temperament trait in sheep.

One of the enriched biological processes considered the term positive regulation of calcium-mediated signaling (GO:0050850, *p*-val = 0.001), where the FSH receptor gene and others (*CD24*, *CDH13*, *SYK*, *LOC101112639*) were observed. The term locomotor behavior (GO:0007626, *p*-value = 0.036) considers the specific movement of an animal from one place to another in response to external or internal stimuli and/or a combination of the internal state and external conditions of that animal. A subset of this GO_term is directly linked to the behavior term (GO:0007610), described as an animal’s responses to internal or external stimuli (actions or inactions), through a mechanism that involves the activity of the nervous system. Another GO_term regulation of ERK1 and ERK2 cascade (Extracellular Signal Regulated Kinase) (GO:0070372, *p*-val = 0.032) participates in various biological responses. This term is associated with several processes that modulate the frequency, rate, or extent of signal transduction mediated by the ERK1 and ERK2 cascade. This signaling cascade is a pathway formed by mitogen-activated protein kinases that regulate a wide variety of cellular processes, as diverse as proliferation, differentiation, survival, and stress [85]. The GO_term linked to carbohydrate metabolic processes (GO:0005975, *p*-val = 0.013) is also revealed by the association that presents the availability of energy to cope with a stressor, specifically the physiological responses to stress that include an increase in metabolic rate and energy, as well as an increase in lipid catabolism and protein degradation. These physiological changes in the animal help to maintain the immediate availability of energy and oxygen [74,86], and are the basis of the “fight or flight” response, the first autonomous response of the individual to face the stressor or to escape from it [87]. This article provides information on SNP and candidate genes linked to the temperament and stress traits in sheep. Given that animals with different temperaments respond differently to stressful situations, the presence of polymorphisms in genes associated with temperament may be associated with the health status of the animals. Therefore, from a clinical point of view, the candidate genes linked to stress and temperament could be of great help when considering the health of animals in productive systems. Since clinical parameters were not evaluated in this study, research is necessary to estimate the implications on the health of the animals, focusing on selection criteria in the prevention of diseases along with productive and behavioral traits.

## 6. Conclusions

In the current study, SNP associated with the temperament trait in sheep were detected. These SNP were not previously reported, which opens a window for exploration, not only linked to temperament traits but also to different metabolic pathways that could have an impact on production and welfare. The genetic variance of the SNP revealed that there are regions of the genome associated with greater variability and that temperament is not regulated by the effect of a major gene, but rather is a multigenic trait. Nine genes were detected in the genomic regions on chromosomes 6, 10, and 21 (genes: *GRID2*, *GPRIN3*, *LOC101110773*, *LOC101110521*, *PYGM*, *SYVN1*, *CAPN1*, *FADS1*, *SYT7*), linked to the energetic activity of the organism, synaptic transmission, meat tenderness, and calcium associated activities. The identification of these genes, metabolic pathways, and their respective functions should contribute to a better understanding of the genetic mechanisms that regulate the temperament trait in sheep. The 5% of the SNP with the greatest effect determined a set of 900 genes linked to various metabolic pathways, where the gene ontology analysis shows that there are several processes linked to the regulation of ATP, calcium, cell activity, locomotion and behavior, lipid and carbohydrate metabolism, modulation of synaptic transmission, among others. Several of these metabolic pathways are of interest for the temperament trait but need further exploration.

## Figures and Tables

**Table 1:** SNP detected within the windows explaining more than 0.5% of the genetic variance, sorted in descending order, associated with temperament in sheep.

rs Code	Pos (bp)	CHR	% Var	Variant Type	Candidate Gene
rs402505013	42,295,749	21	1.4691	Splice acceptor var	*PYGM*
rs419347404	42,601,748	21	1.4519	Intron variant	-
rs161627521	42,654,067	21	1.3288	Intron variant	*SYVN1*
rs413708295	42,714,381	21	1.1617	Intron variant	*CAPN1*
rs421553713	42,714,613	21	1.0906	Intron variant	*CAPN1*
rs161627624	42,715,850	21	0.8537	Synonymous variant	*CAPN1*
rs408317317	29,353,089	10	0.7803	Intron variant	*LOC101110773*
rs428995675	29,072,930	10	0.7738	Intron variant	*LOC101110521*
rs407693533	35,491,698	6	0,7722	Intron variant	-
rs422603241	31,453,177	6	0.7688	Intron variant	*GRID2*
rs422288687	29,304,176	10	0.7678	Intron variant	*LOC101110773*
rs427220269	29,054,709	10	0.7670	Intron variant	*LOC101110521*
rs421383362	29,188,403	10	0.7636	Intron variant	*LOC101110521*
rs409829992	29,162,222	10	0.7624	Intron variant	*LOC101110521*
rs399382510	29,202,499	10	0.7619	Intergenic variant	-
rs411759303	35,511,497	6	0.7430	Missense variant	*GPRIN3*
rs400430030	29,421,760	10	0.6745	Intron variant	*LOC101110773*
rs419116702	29,030,595	10	0.6646	Intron variant	*LOC101110521*
rs398879843	35,191,867	6	0.6423	Intergenic variant	-
rs161618576	39,638,962	21	0.6420	Downstream gene variant	-
rs424142667	35,511,899	6	0.6415	Missense variant	*GPRIN3*
rs410592527	35,184,703	6	0.6228	Intergenic variant	-
rs408222545	39,642,723	21	0.6204	Upstream gene variant	-
rs419203432	29,415,140	10	0.6173	Intron variant	*LOC101110773*
rs161618641	39,646,260	21	0.5915	Upstream gene variant	-
rs416558978	35,254,368	6	0.5873	Intergenic variant	-
rs417392501	39,651,749	21	0.5832	Downstream gene variant	-
rs404318469	42,719,476	21	0.5826	Intron variant	*CAPN1*
rs403363266	39,653,383	21	0.5657	3 prime UTR variant	*FADS1*
rs419203432	29,415,140	10	0.5595	Intron variant	*LOC101110773*
rs403382566	35,512,106	6	0.5508	Missense variant	*GPRIN3*
rs421709693	39,432,569	21	0.5434	Intergenic variant	*SYT7*
rs427110197	39,654,860	21	0.5384	Intron variant	*FADS1*
rs398157763	29,455,959	10	0.5363	3 prime UTR variant	*LOC101110773*
rs399480023	31,217,615	6	0.5249	Intron variant	*GRID2*
rs399060511	35,275,766	6	0.5247	Intergenic variant	-
rs406335698	35,276,244	6	0.5219	Intergenic variant	-

rs = SNP reference; bp = base pairs, position of the SNP in the reference genome (version oar_v3.1); CHR = chromosome; % Var = percentage of the additive genetic variance for each SNP; (-) = no candidate gene was detected for the SNP position.

**Table 2:** Genomic regions of chromosomes with adjacent SNP explaining more than 0.5% of the additive genetic variance and candidate genes for temperament trait.

Chromosome	Start Position (bp)	End Position (bp)	Candidate Genes
6	35,138,403	35,512,106	*GRID2*, *GPRIN3*
10	29,030,595	29,455,959	*LOC101110773*, *LOC101110521*
21	39,432,569	42,419,476	*PYGM*, *SYVN1*, *CAPN1*, *FADS1*, *SYT7*

bp = base pairs.

**Table 3:** Detected pathways from the list of captured genes with the top 5% SNP effect and their classification into 4 classes associated with the *p*-value of enrichment.

Pathways	*p*-Value
**Signaling**	
Phosphatidylinositol signaling system	0.0042
Adherens junction	0.0094
Phoaphopase D signaling pathway	0.0240
Pathways in cancer	0.0310
Axon guidance	0.0370
Calcium signaling pathway	0.0480
**Metabolism**	
Glycosaminoglycan biosynthesis—heparin sulfate/heparin	0.0070
ECM—receptor interaction	0.0270
Metabolic pathway	0.0340
**Steroidogenesis**	
Ovarian steroidogenesis	0.0170
**Others**	
Arrhythmogenic right ventricular cardiomyopathy	0.0370

## Data Availability

Restrictions apply to the availability of these data. Data were obtained by INIA and are available from the authors with INIA’s permission.
